# The Importance of Appropriate Diagnosis in the Practical Management of Chronic Obstructive Pulmonary Disease

**DOI:** 10.3390/diagnostics11040618

**Published:** 2021-03-30

**Authors:** Naozumi Hashimoto, Keiko Wakahara, Koji Sakamoto

**Affiliations:** Department of Respiratory Medicine, Nagoya University Graduate School of Medicine, Nagoya 466-8550, Japan; wakahara@med.nagoya-u.ac.jp (K.W.); sakakoji@med.nagoya-u.ac.jp (K.S.)

**Keywords:** chronic obstructive pulmonary disease, spirometry, lower limit of normal

## Abstract

Chronic obstructive pulmonary disease (COPD) is projected to continue to contribute to an increase in the overall worldwide burden of disease until 2030. Therefore, an accurate assessment of the risk of airway obstruction in patients with COPD has become vitally important. Although the Global Initiative for Chronic Obstructive Lung Disease (GOLD), the American Thoracic Society (ATS) and European Respiratory Society (ERS), and the Japanese Respiratory Society (JRS) provide the criteria by which to diagnose COPD, many studies suggest that it is in fact underdiagnosed. Its prevalence increases, while the impact of COPD-related systemic comorbidities is also increasingly recognized in clinical aspects of COPD. Although a recent report suggests that spirometry should not be used to screen for airflow limitation in individuals without respiratory symptoms, the early detection of COPD in patients with no, or few, symptoms is an opportunity to provide appropriate management based on COPD guidelines. Clinical advances have been made in pharmacotherapeutic approaches to COPD. This article provides a current understanding of the importance of an appropriate diagnosis in the real-world management of COPD.

## 1. Introduction

Chronic obstructive pulmonary disease (COPD) is projected to continue to contribute to an increase in the worldwide burden of disease until 2030 [1]. Therefore, an accurate assessment of the risk of airway obstruction in patients with COPD has become vitally important. The Global Initiative for Chronic Obstructive Lung Disease (GOLD), the American Thoracic Society (ATS) and European Respiratory Society (ERS), and the Japanese Respiratory Society (JRS) all provide criteria with which to diagnose COPD [2,3,4]. Although the clinical guideline, an official statement of the American College of Physicians (ACP), American College of Chest Physicians (ACCP), ATS, and ERS, does not recommend the evaluation of airflow limitations by respiratory function testing in patients without respiratory symptoms [2], many studies suggest that COPD is under-diagnosed.

Smoking exposure also causes several systemic comorbidities in COPD patients [5]. Although severe acute respiratory syndrome coronavirus 2 (SARS-CoV-2), the virus responsible for the coronavirus disease 2019 (COVID-19) pandemic, has infected over 100.0 million people around the world and caused more than over 2.2 million deaths [6], there is mounting evidence that COPD may be a risk factor for more severe COVID-19 disease [7]. Furthermore, the coexistence of several comorbidities hinders efforts to illuminate the pathogenesis of COPD and the heterogeneity of disease progression. Mounting evidence suggests that the heterogeneity of disease progression in COPD might be due to the varying lung function trajectories [8,9].

Although, there are many critical issues for COPD-related outcomes in the real-world clinical settings, understanding whether insufficient management arising from undiagnosed COPD might affect the outcomes related to COPD has not been concisely reviewed, including COPD management (acute exacerbation, hospitalization, and mortality), development of systemic comorbidities (cardiovascular diseases and frailty), the coronavirus disease 2019 (COVID-19) pandemic, and postoperative management of resected lung cancer patients [10,11].

This article provides the current understanding of appropriate diagnosis in the real-world management of COPD.

## 2. Method

The aim of this study was to evaluate the clinical issues in undiagnosed COPD patients. PubMed was searched for population-based estimates published during the period 1980–2020. The search terms included ‘‘chronic obstructive pulmonary disease’’ and either “undiagnosis”, “emphysema”, “lower limit of normal”, “prevalence”, “exacerbation”, “systemic diseases”, “comorbidity”, “coronavirus disease 2019”, “post-operative complication”, or “mortality”. Articles were included if they: (1) Provided the total population for evaluating the clinical issues in undiagnosed COPD patients; and (2) gave sufficient method details to evaluate the outcomes using the criteria defined by the investigators. Studies that might not provide any conclusion on the issues were excluded. From the 436 studies, which the initial search identified, 61 articles were selected to achieve our aim for this review.

## 3. Results

### 3.1. Prevalence of COPD

World-wide cohort studies suggest that the prevalence of COPD is estimated to be about 10% to 14% [12,13]. Under the Japanese Industrial Safety and Health Act, a chest X-ray examination is included in a legal medical examination. It aids the early detection of lung cancer, as a lung cancer screening method. An evaluation of airflow limitation by spirometry examination is optional in statutory medical examinations in Japan. For the early detection of COPD, some studies evaluated airflow limitation by spirometry among subjects aged 40 years or older who had participated in community-based annual health checks in Japan [14,15]. The prevalence of COPD in adults, aged 40 and over, is estimated to be around 10% in Japan.

Although the prevalence of lung cancer is estimated to be about 3 % in COPD patients [16], the coexistence rate of COPD was more than 40% in resected lung cancer patients [17]. A therapeutic option, other than surgery, such as chemotherapy and/or radiation, might be selected for the older lung cancer patients with COPD [17,18,19]. To determine the substantial prevalence of COPD among Asian patients with newly diagnosed lung cancer who were sequentially registered, spirometry was performed when applying bronchoscopy for lung cancer diagnosis. The coexistence rate of COPD was 54.4% among 270 new lung cancer cases that underwent bronchoscopy, and 84.4% were diagnosed with COPD for the first time [20]. In the study population, 61.3% of men had COPD, but only 35.2% of women had COPD. In addition, 95.5% of men had a history of smoking, whereas 67.6% of women were non-smokers. The percentage of non-smokers among women with lung cancer was only 10.5% in a study by Loganathan et al. [21]. The discrepancy might be due to difference in smoking habits in each country. As the severity of airflow limitation was evaluated in these patients with COPD, the proportion of grade 1 (mild) and grade 2 (moderate) in the GOLD classification was more than 90 % of COPD cases. [20]. When the association between airflow limitation and thin-section computed tomography (TSCT)-determined emphysema was evaluated, 38.8% of subjects with airflow limitation were found to have TSCT-determined emphysema. Airflow limitation was observed in 68.1% of subjects with TSCT-determined emphysema [22]. It was also reported that the use of deep residual networks on chest CT scans for ex-smokers, and current smokers who underwent lung cancer screening, was an effective case-finding method in detecting and diagnosing COPD [23].

### 3.2. Clinical Significance of COPD Diagnosis

#### 3.2.1. Management of COPD and Its Exacerbations

The Evaluation of COPD Longitudinally to identify the Predictive Surrogate End-points (ECLIPSE) study shows that a COPD acute exacerbation event can itself predict the next COPD acute exacerbation event in a COPD acute exacerbation risk assessment in patients with COPD GOLD grade 2 or higher [24]. Nevertheless, more than 75% of individuals with COPD, who have been symptomatic for at least five years, are not diagnosed [25]. Although more than 50% of Asian patients with newly diagnosed lung cancer had COPD, only 8.5% of the total study population had been diagnosed and managed as having COPD [20]. This finding is compatible with a retrospective analysis of a clinical cohort in the United Kingdom, which showed that of the COPD patients who had received a chest radiography in the two years before COPD diagnosis, only 33% had spirometry [26].

#### 3.2.2. COPD and Systemic Diseases

Smoking is strongly associated with the development of chronic lung inflammation and systemic inflammation via the inflammatory mediators derived from smoking-stimulated lung tissue [5]. A recent study reported that people with accelerated FEV1 decline were at greater risk of cardiovascular disease, compared to those without accelerated decline over a more than 15-years [27]. Another study suggested that frequency of COPD exacerbation and increasing dyspnea, not a decline in FEV1, might be associated with increasing cardiac events [28]. Frailty is defined as a progressive physiological decline in multiple organ systems marked by loss of function, loss of physiological reserve, and increased vulnerability to disease [29]. Many chronic diseases are associated with frailty and functional decline in older people [30]. The prevalence of frailty might be assumed to be 6–10% in elderly patients with COPD [31,32]. More than 20% of frail elderly subjects with dyspnea might be detected using a near-home screening strategy [33]. Kennedy, et al. demonstrated that frail COPD participants reported significantly worse disease-specific symptoms and the overall quality of life [32]. Furthermore, frailty triggered by worsening of COPD might decrease the time to first hospitalization and an increased the duration of hospitalization [32]. A further understanding of how the COPD frailty phenotype can be modified or treated is warranted.

#### 3.2.3. The Effect of COPD on the Severity and Mortality of COVID-19

For cohorts in China, America, and Italy reporting on hospitalized COVID-19 patients, the prevalence of COPD has ranged from zero to 15% [7,34]. Comparing its severity among COPD patients, the risk of development of severe COVID-19 disease might be relatively lower than the risk among asthma patients, possibly due to the use of inhaler corticosteroid (ICS) [35]. Nevertheless, a recent observational study did not support the hypothesis that regular ICS use might protect against COVID-19-related death among people with asthma or COPD [36]. A decrease in physical activity might be strongly associated with mortality in COPD patients [37]. The extensive social distancing policies and restrictions brought about by the COVID-19 pandemic often makes it difficult for individuals to visit with their physicians, resulting in fewer opportunities to receive pulmonary rehabilitation programs [34].

#### 3.2.4. The Effect of COPD Co-Existence in Resected Lung Cancer

**Figure 1:**
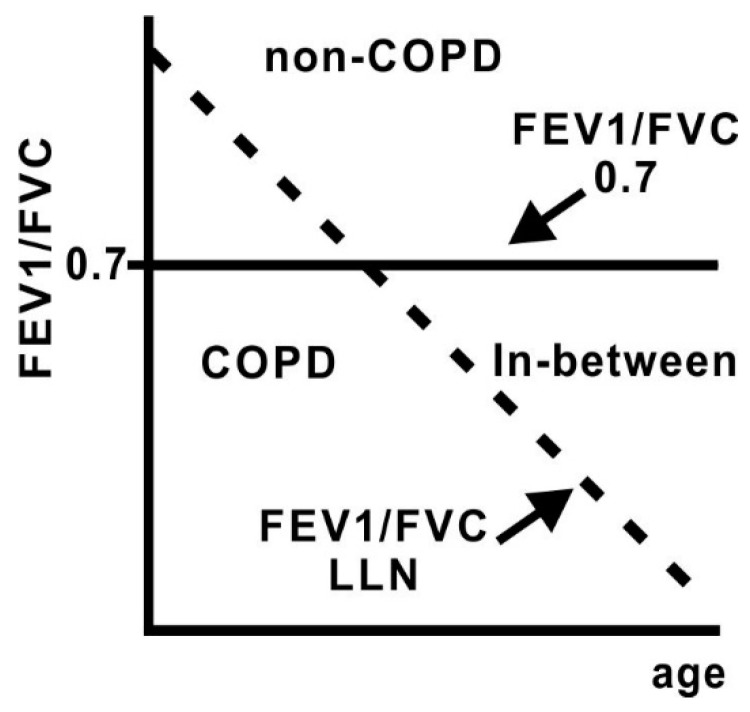
Diagram depicting the fixed 0.7 ratio of FEV1/ FVC and the decline of the LLN of FEV1/ FVC with aging. Modified from [42]. Solid line: The fixed 0.7 ratio. Dotted line: The LLN of FEV1/ FVC with aging.

Most COPD patients with resected lung cancer show no or few COPD-related symptoms, due to mild airflow limitation [20]. Nevertheless, COPD patients with an FEV1/ forced vital capacity (FVC) ratio below 0.70 had a prolonged postoperative stay, and a greater need of prolonged oxygen therapy, than patients without COPD [17,38]. Although clinical guidelines recommend spirometric assessment to evaluate the optimum selection of surgical procedures, in view of the risks of mortality and post-operative complications [11,18], better risk stratification for post-operative outcomes in patients, with COPD undergoing thoracic surgery, has not been fully determined. An FEV1/FVC ratio below the lower fifth percentile of a large healthy reference group (that is, the statistically defined lower limit of normal [LLN]) is used to classify airflow obstruction [39,40]. Studies have not evaluated whether an FEV1/FVC ratio below 0.70 but above the LLN (an “inbetween” group) could identify patients at risk of adverse COPD-related clinical outcomes had not been fully evaluated (Figure 1) [41,42]. When the combined assessment of the 0.70 fixed ratio and the LLN of the FEV1/FVC ratio was used for risk stratification, the in-between group classified by a FEV1/FVC ratio below 0.70, but above the LLN included patients with mild cases of COPD patients. The LLN assessment of the FEV1/FVC ratio might provide more accurate risk stratification in COPD patients undergoing thoracic surgery [41]. In this study, the LLN of FEV1 and FVC were calculated by using the reference equations of the National Health and Nutrition Survey III (NHANESIII), due to the absences of a Japanese reference equation to calculate the LLN of FEV1/FVC [41]. A recent study suggested that the locally derived LLN criteria seem to be better at identifying high-risk individuals with COPD, compared with the LLN criteria from other regions. Whereas, COPD individuals determined by the five different LLN criteria showed similar risk of COPD exacerbations and mortality [43]. Okada, et al. stratified the risk for postoperative outcomes in COPD patients with resected lung cancer by using renewed Japanese spirometric reference variables [3,44]. The studies demonstrated that airflow obstruction, determined by a different lung function reference, had a similar risk of post-operative outcomes [41,44],

#### 3.2.5. The Effect of COPD Coexistence in Advanced Lung Cancer Cases

High COPD coexistence rates were shown in lung cancer cases [20], but no increase in chemotherapy-related adverse events was observed in COPD patients that underwent chemotherapy for advanced lung cancer [19,45]. Therefore, the effect of COPD on patients with lung cancer might depend on treatment options, such as surgery or chemotherapy.

### 3.3. Early COPD

In some cases, COPD develops from accelerated lung function decline. Other cases do not achieve the expected maximally attained lung function in early adulthood, resulting in COPD [8]. Therefore, among COPD patients, those with accelerated disease progression should be identified. Martinez, et al. suggested that a classification to distinguish between “early disease” and late “mild disease” was warranted, in order to aid individualized interventions and modify progression before irreversible damage [9]. They proposed that early COPD should be defined in individuals under 50 years of age with 10 or more pack-years of smoking history and any of these abnormalities: (1) Early airflow limitation (post-bronchodilator FEV1/FVC, LLN); (2) compatible CT abnormalities, and; (3) a rapid decline in FEV1 (> 60 mL/year); that is, accelerated relative to FVC [9,46]. Based on the definition of early COPD [9,46], a recent study demonstrated that the operational definition for early COPD may be effective in excluding individuals unlikely to develop clinical COPD later in life [47]. Furthermore, COPD that develops through a normal maximally attained FEV1 trajectory is associated with increased risks of both respiratory disease mortality and all-cause mortality, compared with COPD that develops through a low maximally attained FEV1 trajectory [48].

## 4. Discussion

In global setting, including Japan, the prevalence of COPD in adults aged 40 and over is estimated to be around 10%. There is a discrepancy of more than 20 times between the approximately five million individuals with COPD and 200,000 patients with COPD undergoing pharmacotherapy [49]. Spirometry was performed to determine the substantial prevalence of COPD among Asian patients with newly diagnosed lung cancer who were sequentially registered. It was found that COPD is a common comorbidity in elderly people with a smoking history [26,46,50].

Many studies point out that undiagnosed COPD patients often visit primary care for COPD-related respiratory symptoms, such as dyspnea, cough, and sputum [25]. This underlines the need for COPD management options [26,46,50]. Primary physicians might not recognize the worsening respiratory symptoms in these undiagnosed patients as involving COPD acute exacerbation. Efficacious COPD management might be provided for patients at an early stage of COPD when patients with respiratory symptoms, who visit primary care, are appropriately diagnosed with COPD by spirometry [51].

Systemic inflammation from smoking-stimulated lung tissue in COPD patients might induce the development of heart disease, osteoporosis, metabolic syndrome, skeletal muscle atrophy, frailty, and depression [5,28,30,31,32,52]. Nevertheless, COPD-related systemic comorbidities, including diabetes, hypertension, and ischemic cardiac disease, might not be associated with the development of post-operative outcomes in resected lung cancer patients [17,38]. Therefore, post-operative outcomes should be recognized as COPD-related outcomes, even in patients with mild cases of COPD. Although, the COPD frailty phenotype might affect COPD-related outcomes, such as respiratory and all-cause mortalities, hospitalization, acute exacerbation, poor quality of life, and depression [32]. It also remains undetermined whether the decreased physical activity in COPD patients, due to the COVID-19 pandemic, could increase the incidence of the COPD-related outcomes. If we can appropriately diagnose COPD, early detection plays a key role in improving the prognosis of patients with COPD [53]. Further investigation is warranted.

There is growing awareness of the need to identify COPD in patients at an early stage [50,53,54,55,56,57]. A recent systematic review suggests that pharmacotherapy is effective in altering the rate of lung function decline and that the annual decline of forced expiratory volume in one second (FEV1) modified by bronchodilators was within the decline, reported for health status and for the exacerbation rate in the clinical trials [58]. Further research efforts are warranted to verify the effectiveness of appropriate management of undiagnosed COPD patients, as mounting evidence suggest early COPD with a rapid decline in FEV1 might be a suitable target for appropriate therapy [47,48].

Two issues remain elusive for providing helpful guidance to the practicing clinicians. Firstly, when should spirometry be performed? Smoking often causes the development of lung cancer and COPD [59,60]. A chest radiography might be examined by primary physicians to screen for lung cancers among patients aged over 70 years and with smoking history [20]. The timing might provide an opportunity to use spirometry tests to detect COPD [20,25,26]. By evaluating the presence of emphysematous lesions on TSCT in individuals under 50 years of age with 10 or more pack-years of smoking history [9], diagnosing emphysema can be an indicator for assessing airflow obstruction [61,62]. Secondly, how should the data from spirometry be evaluated? The combined assessment of the 0.70 fixed ratio and the LLN of the FEV1/FVC ratio could identify patients at risk of adverse COPD-related clinical outcomes in resected lung cancer patients [41,42,44]. Although adult smokers, suspected of having COPD, were reported to be at no increased risk of respiratory morbidity or all-cause mortality until the ratio falls below the age-corrected LLN (even though it is below 0.70) [40], the combined assessment of the 0.70 fixed ratio and the LLN of the FEV1/FVC ratio might provide more accurate management in early COPD patients that develop through a low maximally attained FEV1 trajectory [48], but also COPD patients at an early disease stage [50,53,54,55,56,57]. Further research efforts are warranted for providing helpful guidance to the practicing clinicians.

## 5. Conclusions

This article provides a current understanding of the unresolved issues that arise from insufficient management of undiagnosed COPD and how they might affect the outcomes related to COPD in the real-world clinical settings. In summary, the importance of an appropriate diagnosis in the real-world management of COPD should be emphasized.
